# Uterine Transplantation: Advances, Challenges, and Future Perspectives

**DOI:** 10.3390/diseases13050152

**Published:** 2025-05-15

**Authors:** Ana Pereira, Flávia Ribeiro, Sandra Soares, Hélder Ferreira

**Affiliations:** 1 School of Medicine and Biomedical Sciences, University of Porto, 4050-313 Porto, Portugal; 2 Department of Gynecology, Minimally Invasive Gynecological Surgery Unit, Centro Materno Infantil do Norte, Unidade Local de Saúde de Santo António, 4099-001 Porto, Portugal

**Keywords:** uterine transplantation, utero-transplantation, uterine infertility, neonatal outcomes, maternal complications, psychological status, challenges, future perspectives

## Abstract

Background: Infertility is a multifactorial condition with medical, psychological, demographic, and economic impacts. Around 3–5% of cases are due to uterine dysfunction. Absolute uterine factor infertility (AUFI) refers to infertility caused entirely by the absence or abnormality of the uterus, which prevents embryo implantation or pregnancy viability. Uterus transplantation (UTx) has emerged as a promising treatment for AUFI and has been successfully performed in over 10 countries. Objectives: This study aims to conduct a systematic review of uterus transplantation, evaluating its efficacy and safety, as well as maternal, neonatal, and long-term outcomes. It also explores current challenges and future directions. Methods: The methodology was registered on the PROSPERO platform. A literature search was performed in January 2025 across PubMed, Web of Science, and Scopus for articles published from January 2002 to December 2024 in English or Portuguese. The query was: “uterus/transplantation AND (pregnancy OR complications OR newborn OR premature OR diseases)”. Study quality was assessed by journal impact factor (IF). Data were analyzed using Microsoft Excel. Results: A total of 10 studies were included: four from Sweden, three from the DUETS group, two from the Czech Republic, and one multi-institutional American study. The UTx success rate was 74.0%; clinical pregnancy rate (CPR) and live birth rates (LBR) per embryo transfer (ET) were 36.3% and 22.0%, respectively. No significant increase in congenital or neurological complications was observed. Adverse psychological outcomes were associated with transplant failure or pregnancy loss. Conclusions: UTx is a promising treatment for AUFI, showing favorable pregnancy and birth outcomes without major fetal or neonatal risks.

## 1. Introduction

Infertility is a common issue that carries not only medical and psychological implications but also demographic and economic consequences [1]. It is estimated that up to 15% of the reproductive age population is infertile, affecting approximately 300,000 Portuguese couples [2,3]. With a multifactorial etiology, around 3 to 5% of diagnosed infertility cases are due to uterine dysfunction [2].

Absolute uterine factor infertility (AUFI) refers to infertility that is entirely attributed to the absence (congenital or acquired) or abnormality (anatomical or functional) of the uterus. Pathophysiologically, these conditions prevent embryo implantation or the completion of a pregnancy with fetal viability [4].

Uterine malformations affect approximately 6.7% of the female population, with 7.3% of these women having congenital AUFI. Among these congenital conditions causing infertility, Mayer–Rokitansky–Küster–Hauser (MRKH) syndrome stands out [5]. Characterized by a congenital anomaly in female genital development, resulting in uterine agenesis and vaginal hypoplasia, this syndrome affects about 1 in 5000 women [6]. On the other hand, hysterectomies performed during reproductive age for medical reasons account for approximately 40% of all AUFI cases, making them the leading cause of acquired AUFI [5]. In fact, various clinical conditions may lead to a hysterectomy, including the treatment of malignant neoplasms, definitive treatment of leiomyomas, or obstetric complications such as placenta accreta or massive postpartum hemorrhage [7].

Thus, for women/couples affected by AUFI, the desire for motherhood comes down to three possibilities: the use of in vitro fertilization (IVF) techniques with gestational surrogacy, adoption, or, in the worst-case scenario, not achieving parenthood at all [5].

Uterus transplantation stands out as a promising solution for treating infertility for these women. In simple terms, this new therapeutic approach involves transplanting a uterus from either a living donor (LD) or a deceased donor (DD) into a woman with AUFI. This restores the missing or non-functioning organ, opening a new window of opportunity for conception attempts [8]. Unlike traditional transplants in medicine, this procedure involves a solid organ transplant that is not lifesaving but solely intended to enable pregnancy for women/couples. As a result, the graft is expected to have a short lifespan (only a few years), with hysterectomy/removal of the graft ideally performed after the completion of reproductive goals. Thus, uterus transplantation is the first transplant carried out with the awareness that it will be removed a few years later [9].

From a technical perspective, uterine transplantation is a multi-step and highly demanding surgical procedure. It begins with the retrieval of the uterus from an LD or a DD [4]. In cases of the LD, recent studies advocate for the use of robot-assisted laparoscopic hysterectomy over traditional laparotomy or standard laparoscopy, as it has been shown to reduce donor morbidity without compromising the graft quality or recipient outcomes [4,10]. The primary objective of the donor surgery is to preserve a uterus with sufficiently long vascular pedicles, typically consisting of the uterine arteries and either the uterine veins or segments of the internal iliac veins [11]. Once retrieved, the graft is flushed and prepared, and its vascular patency is confirmed on the back table [4,11]. In the recipient, the graft is placed orthotopically, and vascular anastomoses—most commonly end-to-side—are performed under magnification to connect the uterine vessels to the recipient’s external or internal iliac vessels [11]. A vaginal anastomosis is then established between the donor cervix and the recipient’s vaginal vault, and the uterus is anchored using the surrounding pelvic ligaments. These surgical steps may require modification depending on anatomical variations identified intraoperatively [4].

Historically, the first uterus transplant was performed in Saudi Arabia in 2000 (publicly reported in 2002). However, three months later, a hysterectomy became imperative due to vascular thrombosis, which led to graft necrosis. Nonetheless, scientific advancements continued, and in 2014, the first baby was born in Sweden following a uterus transplant from an LD in a woman with MRKH syndrome. Three years later, the same milestone was achieved for the first time using a DD in Brazil. In July 2023, in the United States of America (USA), the birth of the first baby from a uterus transplant performed outside the scope of clinical trials was announced. Today, uterus transplantation has been successfully performed in more than 10 countries (Sweden, USA, Brazil, Serbia, India, Czech Republic, China, Lebanon, Germany, Italy, Spain, and France). In Portugal, this surgical procedure has not yet been performed [8,12].

Given the significant relevance of this topic today and the many recent advancements in the field, this study aims to conduct a systematic review of uterus transplantation. The goal is not only to synthesize scientific evidence regarding the efficacy and safety of this revolutionary option for treating AUFI but also to examine maternal and neonatal outcomes and identify challenges and future perspectives.

## 2. Materials and Methods

The methodology of this systematic review was registered on the PROSPERO platform (ID: CRD420250604888) to ensure methodological transparency and to prevent bias and duplication of similar studies. Additionally, this study was designed following the PRISMA (Preferred Reporting Items for Systematic Reviews and Meta-Analyses) 2020 guidelines (see Supplementary Materials) [13,14].

Regarding the search strategy, it included articles available in the PubMed (Medline), Web of Science, and Scopus databases. The search was conducted in January 2025, with a time frame limited to studies published between January 2002 and December 2024. Only articles written in English or Portuguese were considered. The query used across all digital platforms was: “uterus/transplantation AND (pregnancy OR pregnancy complications OR infant, newborn, diseases OR infant, premature OR complications)”. The article selection method aimed to answer the following PICO question: what is the success rate (transplantation, pregnancy, live births), maternal and neonatal complication rates, challenges, and future perspectives of uterus transplantation in women with AUFI?

Concerning the inclusion criteria, this systematic review considered peer-reviewed clinical studies (observational studies, retrospective and prospective studies, clinical trials, and cohort studies) on uterus transplantation that reported success rates (transplantation, pregnancy, and term or preterm live births (LBs)), maternal outcomes, or neonatal complications. Conversely, preclinical or animal studies, isolated case reports, editorials, and opinion pieces were excluded from the analysis. Studies lacking relevant data or having poor methodological quality, duplicates, or systematic and narrative reviews were also eliminated. Additionally, articles discussing ethical concerns, surgical techniques, donor and recipient selection criteria, immunosuppression protocols, and donor-related outcomes were excluded.

After applying the aforementioned methodology, article titles and abstracts were screened, and studies with potential relevance for inclusion in this systematic review were selected. Subsequently, information such as title, author, year of publication, country, publication platform, DOI, peer review status, study design, participant characteristics, interventions, assessed outcomes, and main results were collected using tools like Microsoft Excel and reference management software such as EndNote.

The quality assessment of the selected studies was performed based on the impact factor (IF) of the journal in which the article was published. Finally, data analysis was conducted using software like Microsoft Excel, calculating statistical means, extremes, and relevant intervals for the critical interpretation and evaluation of the collected information.

## 3. Results

**Figure 1:**
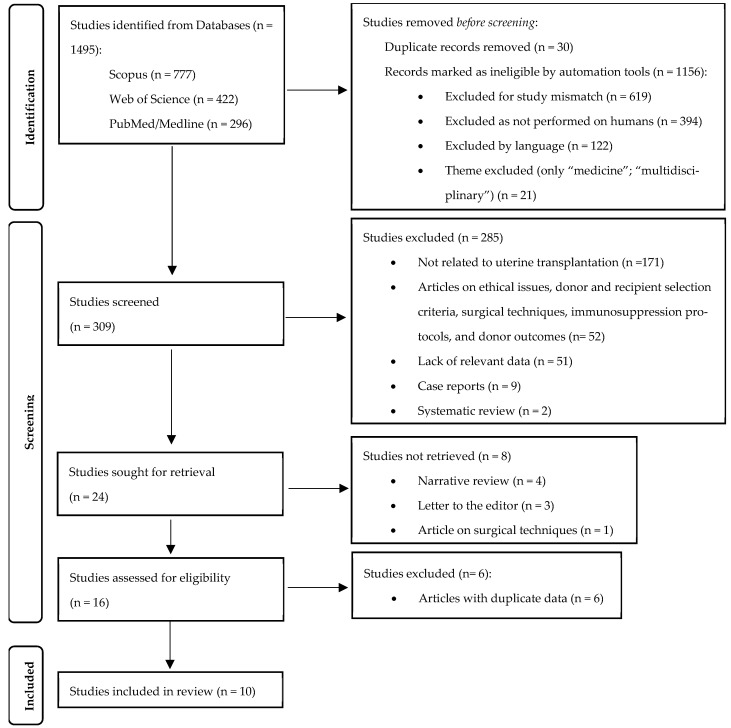
Flowchart adapted from PRISMA 2020 [14], summarizing the study selection process.

Using the previously described search strategy, a total of 1496 articles were identified (PubMed/Medline: 296; Web of Science: 422; Scopus: 777). In the first phase, 1186 articles were removed (duplicates: 30; automatically excluded: 1156). From the remaining 309 articles, 24 studies were sought for retrieval, of which only 16 were assessed for eligibility, and 10 were ultimately included in this systematic review (Figure 1). Among these, four were clinical trials conducted in Sweden [9,11,15,16], three belonged to the Dallas Uterine Transplant Program, USA (DUETS) [17,18,19], two contained data from the Czech Republic [20,21] and one was a multi-institutional American study that included data from Dallas, Philadelphia, and Cleveland [22]. The main characteristics of the included studies are presented in Table 1. To avoid data duplication, and because the publication date of the overlapping data between the DUETS articles and the American multi-institutional study was the same (September 2024), DUETS data were extracted from the multi-institutional article, leaving only information from Philadelphia and Cleveland separately.

The results are categorized into uterine transplant success rates and complications (Table 2), pregnancy success rates and obstetric complications (Table 3), neonatal and infant outcomes (Table 4), and the psychological status of recipients (Table 5).

### 3.1. Transplant Success Rate and Complications

**Figure 2:**
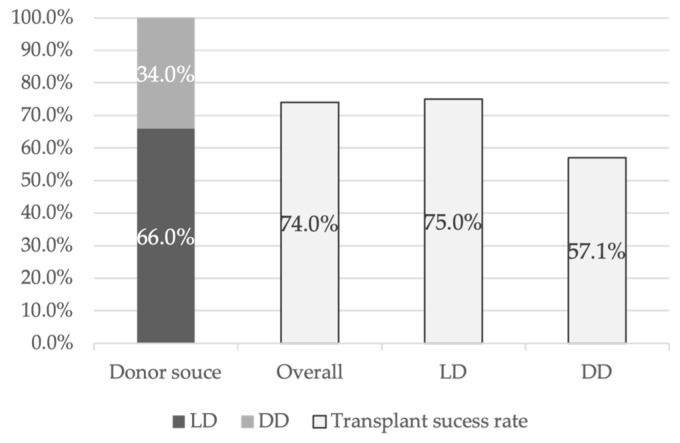
Donor source and transplant success rate.

This systematic review included 50 women/recipients, of whom 94.0% had MRKH syndrome and 6.0% had undergone a hysterectomy. The mean age ranged from 28 to 31.5 years, with the youngest recipient being 20 years old [17] and the oldest being 38 years old [11]. Of the 50 transplants performed, 66.0% of the uteri came from LDs, while the remaining 34.0% were from DDs (Figure 2). Uterine transplant success was defined by the establishment of regular menstruation for at least 6 months post-transplant. Within this cohort, the uterine transplantation success rate was 74.0% (Figure 2), while the graft failure rate was 26.0%. A more detailed statistical analysis, considering only articles from DUETS, Sweden, and the Czech Republic [11,17,21], showed that 75.0% of transplants from LDs were successful, whereas the success rate for transplants from DDs was 57.1% (Figure 2). Regarding graft failure causes, 72.7% of cases were due to anastomotic issues or arterial/venous thrombosis. Additionally, one graft failure was attributed to hemorrhagic shock, another to persistent uterine infection, and one due to chronic rejection.

Regarding acute graft failure, 28 women were evaluated [11,17,21], with 28.6% experiencing no acute rejection episodes, 35.7% experiencing one episode, and 32.1% having at least two episodes. The most reported complications were infections, vaginal stenosis, renal toxicity (induced by calcineurin inhibitors), and cytopenia. Infections included urinary tract infections (UTIs), cytomegalovirus (CMV) infections, fungal infections, surgical incision infections, herpes infections, pelvic abscesses, sinus infections, and *Clostridium difficile* colitis. Approximately 44.4% of the evaluated women [11,17,21] had an infection episode, one of which resulted in a hysterectomy due to graft failure [11]. Furthermore, considering women who had a successful uterus transplant [17,21], vaginal stenosis was reported in 71.4%, renal toxicity occurred in 14,3% and cytopenia were observed in 57.1%.

### 3.2. Pregnancy Success Rate and Obstetric Complications

Another critical outcome to assess the effectiveness of uterine transplantation is the success of pregnancy and the ability of transplanted women to carry an LB.

Keeping in mind that IVF cycles are performed prior to the transplant and frozen embryos are available for transfer once a successful transplant is confirmed, the average time between transplantation and the first embryo transfer (ET) ranged from 125 to 390 days [17,21,22], with the shortest interval being 64 days [17] and the longest of 551 days [21].

**Figure 3:**
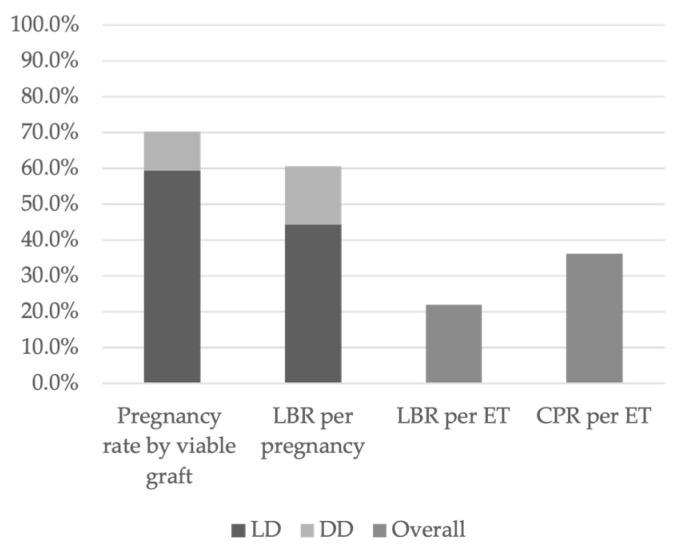
Pregnancy rate, LBR, and CPR.

According to data from DUETS, Sweden, and the Czech Republic, 70.3% of women with a viable transplanted uterus achieved at least one clinical pregnancy. Among them, 84.6% had received a uterus from an LD, while 15.4% had received one from a DD (Figure 3) [16,17,21].

**Figure 4:**
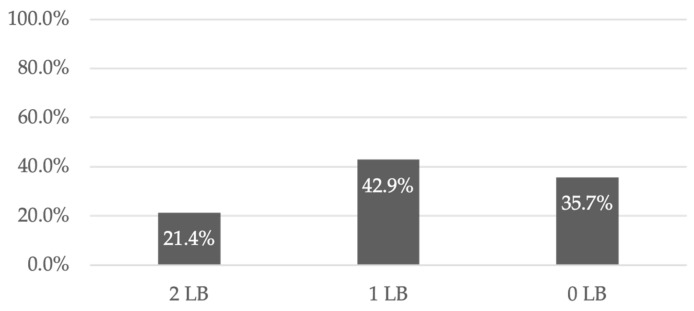
LB per recipients.

Overall, 60.7% of clinical pregnancies resulted in LB, of which 73.0% originated from LD transplants and 27.0% from DD transplants (Figure 3). The clinical pregnancy rate (CPR) per ET was 36.3%, while the live birth rate (LBR) per ET was 22.0% (Figure 3) [16,17,21,22,23]. Approximately 34.6% of women with a clinical pregnancy experienced at least one pregnancy loss [16,17,21]. However, 21.4% of recipients had two LBs, 42.9% had one LB, and 35.7% did not achieve a successful LB (Figure 4) [16,21].

Regarding obstetric complications, cases of gestational hypertension (HTN), gestational diabetes, preeclampsia (PE), preterm labor, intrahepatic cholestasis of pregnancy, cervical insufficiency, placentation abnormalities (placenta previa and placenta accreta), subchorionic hematoma, and vaginal hemorrhage were reported. The most common complications were PE (15.4%) and preterm labor (15.4%), followed by gestational HTN and diabetes, each affecting 11.5% of women. It is worth mentioning that there were two cases of intrahepatic cholestasis, two cases of cervical insufficiency requiring cerclage, and two cases of placentation abnormalities. Lastly, one case of subchorionic hematoma led to vaginal hemorrhage [16,17,21].

### 3.3. Neonatal and Infant Outcomes

Assessing neonatal and infant outcomes is essential to evaluate fetal, neonatal, and infant safety, as well as to identify potential concerns among children born following uterine transplantation.

The average gestational age ranged between 35 weeks (W) and 3 days (d) and 37 W and 0 d, with the shortest gestation being 30 W and 6 d [17] and the longest being 38 W and 1 d [24]. Approximately 60.7% of newborns (NBs) were preterm, while 39.3% were full-term [16,17,21]. Among the 16 evaluated LBs, 87.5% were from LD recipients, and 12.5% were from DD recipients [18,21]. All neonates were delivered via cesarean section [16,17,21], with a mean Apgar score of 8 at the first minute and 9 at the fifth minute, ranging from 4 to 10 at the first minute and from 8 to 10 at the fifth minute [19,21].

Among the NBs evaluated, 78.6% (*n* = 11) had no apparent congenital anomalies. The remaining 21.4% included three cases: one with a patent foramen ovale, one with clitoromegaly (spontaneously resolved) associated with anterior urethral displacement (surgically corrected at 11 months), and one with a helical ear deformity. Other neonatal complications included six cases of respiratory distress syndrome (RDS), two cases of neutrophilia with negative blood cultures, two cases of neonatal hypoglycemia, and one case of fetal lung fluid retention. It is also important to mention that subtherapeutic levels of tacrolimus were reached at the end of 5/6 days of life [19,20].

At up to 2 months of age, 100% (*n* = 12) of the evaluated infants exhibited no alterations in anthropometric data (weight, height, and head circumference) or neurodevelopmental disorders. At the 6-month assessment, 100% (*n* = 13) of the infants presented normal anthropometric data. However, at the 12-month assessment (12 children evaluated), one child was classified with abnormal anthropometric parameters, and another did not exhibit adequate neurodevelopment for postnatal age, as they were unable to use the words “father” or “mother” with meaning—this issue was resolved in a follow-up consultation. At the 18-month assessment, nine children were evaluated. Among them, three were classified as having abnormal anthropometric data, and three exhibited mild cognitive deficits; one child was unable to say six words or point to a body part (improved in a follow-up consultation); another had ongoing developmental delay (referred to a speech therapist); and the third was unable to say six words (improved in both quantity and quality of communication skills). However, at the 2-year assessment, four out of five children reached the expected anthropometric and neurological development milestones. Only one child showed a persistent delay in communication skills, being unable to say more than 20 words or form sentences or expressions—this child was referred for speech therapy twice a week. It is also worth noting that no child required hospitalization for infectious reasons. In summary, the physical, neurological, and immunological developmental milestones were, overall, appropriate for age in the first 2 years of life. The cognitive deviations observed were mild and temporary, improving with the interventions performed. There was no higher rate of developmental delay among preterm-born children, nor was there a correlation between previous rejection episodes and developmental delay [18].

At the 3-year assessment, 100% (*n* = 2) of the children were within the expected anthropometric parameters. Notably, one case of idiopathic thelarche emerged at 21 months. Additionally, one child exhibited a mild cognitive deficit in expressive communication [20].

### 3.4. Psychological Status of the Recipients

At the time of inclusion, 100% of recipients indicated good quality of life (QoL) in the physical component (physical component summary—PCS) of the Short Form Health Survey-36 (SF-36) instrument, while three recipients did not meet the good QoL score in the mental component (mental component summary—MCS) of the same instrument. No recipient reported significant symptoms of anxiety or depression when completing the Hospital Anxiety and Depression Scale (HADS). Regarding the application of the Dyadic Adjustment Scale (DAS) and the Fertility Quality of Life Tool (FertilQol) questionnaires, 100% of couples reported a good level of marital adjustment and good infertility-related QoL, respectively [15].

Three months after the study began, recipients with ongoing grafts exhibited significant stress, decreased physical functioning, and increased bodily pain (SF-36). At the end of one year, it was observed that couples who underwent uterine transplantation had not only lower levels of anxiety and depression (HADS) but also better infertility-related QoL (FertilQol) compared with couples undergoing IVF for the first time. At this point, 100% of couples maintained a good level of marital adjustment (DAS) [9].

In the second-year assessment, five recipients did not achieve good QoL scores in PCS or experienced a decline of more than 10 points (one standard deviation): one recipient had graft failure, two had pregnancies resulting in LBs, and two were in the early stages of pregnancy. In the mental component (MCS) assessment, the number of recipients who did not reach the minimum score for good QoL increased to five. In the HADS questionnaire, two recipients expressed significant symptoms of anxiety and depression: one recipient had graft failure, and another had no LB. Finally, it is worth mentioning that, during this period, one couple divorced, resulting in poor marital adjustment levels for this pair (DAS) [15].

In the third year, the results of the PCS questionnaire were comparable with the baseline assessment. The number of recipients with poorer MCS and HADS classifications decreased to four and one, respectively. The DAS assessment remained similar to the previous year [15].

Finally, in the fourth year, 100% of the evaluated women indicated good QoL in both the physical and mental components of the SF-36 as well as a good level of marital adjustment (DAS) [16].

## 4. Discussion

Uterus transplantation is a relatively recent treatment option for women/couples diagnosed with AUFI. Given the many advancements in recent years, it is essential to reflect on its safety and efficacy by evaluating transplant success rates and associated complications, pregnancy success rates and related obstetric complications, as well as the psychological status of recipients. Furthermore, because the primary goal of this technique is achieving LBs, it is also crucial to assess the fetal and infant implications of this procedure.

### 4.1. Uterine Transplant Success Rates and Complications

Comparing the results of uterus transplantation with those of other solid organ transplants is neither straightforward nor entirely linear. In most cases, solid organ transplants are life-saving procedures, and graft and recipient survival are often measured in years. However, these are not standard markers in uterus transplantation, where the primary goal is to achieve a functioning graft capable of sustaining an LB. Despite these differences, uterus transplantation success and graft failure rates are not far from those observed in liver transplantation if we compare the same time intervals, considering the beginning of research on this type of technique. The first liver transplant was performed in 1963 by Starzl et al. [25]. According to a study evaluating nearly 4000 liver transplant recipients between February 1981 (18 years after the first liver transplant) and April 1998, the overall graft survival rate was 70% in the first year [26]. Therefore, the uterus transplant success rate of 74.0% calculated in this systematic review presents highly promising results for this novel surgical technique. Notably, several authors of the included studies reported an improvement in graft survival rates over time, which is an encouraging finding [17]. Furthermore, differences exist depending on whether the uterus came from an LD or a DD. Transplants from LDs showed higher success rates, a trend also observed in other solid organ transplants. The 2023 annual report from the Scientific Registry of Transplant Recipients (SRTR) highlights that 5-year graft survival rates for kidney transplants range between 80.2% and 90.0% for LD kidneys, compared with 66.1% to 82.2% for DD kidneys [27]. Finally, comparing uterus transplant outcomes with those of other non-life-saving transplants, such as face, hand, or arm transplants, is not pertinent, as these are also recent techniques and are still in an early experimental phase, preventing a reliable comparison of outcomes [28,29].

Given that nearly 75% of graft failures resulted from anastomotic problems or arterial/venous thrombosis, a key challenge for the future will be refining surgical techniques and researching improved antithrombotic protocols.

From surgical risks (transplantation and cesarean section) to immunosuppression-related complications, uterus transplant recipients face several potential risks. The most common maternal complication observed was vaginal stenosis. According to some authors, pre-transplant vaginal type (native, dilated, or L-Vecchietti procedure) did not seem to influence the incidence of post-transplant vaginal stenosis, which itself did not appear to interfere with pregnancy conception [21].

Another frequently observed risk was infection (44.4%). However, interpreting this finding is complex, as “infection” encompassed multiple clinical conditions with various risk factors, ranging from the surgical procedure itself to the immunosuppressive state of the patients. More detailed studies are needed to determine the actual infection risk and identify the most significant contributing factors to reduce infectious complications associated with uterus transplantation.

Regarding renal toxicity induced by calcineurin inhibitors (vasoconstrictive effect), it is important to consider that approximately 30% of women with MRKH syndrome have congenital renal anomalies alongside uterine agenesis. This increases the risk of renal toxicity and injury in these patients. Therefore, long-term studies are necessary to evaluate the renal impact of immunosuppressive therapy in uterus transplant recipients [30]. Additionally, further research is needed to assess the long-term effects of immunosuppression, particularly because these women are otherwise healthy and do not require transplantation or immunosuppression for survival [17].

### 4.2. Pregnancy Success Rates and Obstetric Complications

Given a functional transplanted uterus, the clinical pregnancy rate (CPR) and live birth rate (LBR) per embryo transfer (ET) (36.3% and 22.0%, respectively) are comparable with or even higher than those observed in cases of infertility due to non-uterine factors, such as endometriosis, polycystic ovary syndrome, or tubal factor infertility (CPR and LBR per ET of 20.4% and 23.4%, respectively) [31]. These findings suggest that once the missing element (uterus) is restored, it can function at a similar level to native uteri in women undergoing IVF for other infertility-related reasons. Moreover, the LBR in uterus transplant recipients surpassed that of women with MRKH syndrome using gestational surrogacy (LBR per ET of 18%) [32]. This indicates that uterus transplantation may offer MRKH patients a greater likelihood of fulfilling their reproductive aspirations compared with surrogacy. Nonetheless, further studies are necessary to identify pre-transplant risk factors that predispose women to recurrent implantation failure [22].

Regarding obstetric complications, the risk of PE in uterus transplant recipients does not appear to be higher than in kidney transplant recipients, where preeclampsia rates can reach up to 42% [33]. In fact, women who undergo other solid organ transplants often have long-term comorbidities, such as renal, hepatic, or cardiac insufficiency, which increase their risk of obstetric complications, including PE [20]. However, uterus transplant recipients remain at higher risk of PE due to immunosuppression and the potential coexistence of renal anomalies, especially in those with MRKH syndrome.

### 4.3. Neonatal and Infant Outcomes

NBs from uterus transplant recipients are conceived through IVF and develop in uteri that are not genetically related to either the mother or the child. These uteri also have altered arterial and venous blood flow, which may influence how blood is distributed in the myometrium. Additionally, these fetuses are exposed to immunosuppressive therapy during at least their organogenesis and fetal development period [18,20].

More than half of these NBs were preterm. However, this was primarily due to early-stage research protocols, where deliveries were scheduled before 37 W. Over time, researchers recognized that early delivery posed unnecessary risks, leading to adjustments in scheduled delivery times beyond 37 W when no contraindications were present [16]. Nonetheless, pregnancies following uterus transplantation may carry an inherent risk of preterm birth due to the increased likelihood of PE and other complications associated with immunosuppression [20].

Neonatal complications observed in these cases are similar to those found in infants born at comparable gestational ages, suggesting no direct link between these complications and uterus transplantation [19]. Additionally, for the authors of the included articles, reported congenital anomalies (patent foramen ovale, clitoromegaly with anterior urethral displacement, and helical ear indentation) did not appear to be related with maternal immunosuppression [18]. Thus, no congenital malformations were directly associated with uterus transplantation.

By 5–6 days of life, fetal tacrolimus levels reached subtherapeutic ranges. However, because this is an organ transplant that will result in the generation of a new being under immunosuppressive doses, it is important to study the minimum immunosuppressive iatrogenesis that must be applied to these women and fetuses so that the objective of the transplant can be achieved.

Regarding the alterations considered about anthropometric data, a better characterization of these deviations is essential to determine whether they are related to uterine transplantation or if they are also common in other conditions, such as prematurity. As for neurodevelopmental outcomes, no significant neurological deviations were observed in the evaluated children. Some authors claim that these results are consistent with other studies conducted on children born to mothers who have undergone other types of solid organ transplants (kidney and liver) or conceived through IVF techniques. The observed neurocognitive deviations in these children are attributed to other factors, such as preterm birth, rather than being directly related to uterine transplantation or immunosuppression [18]. However, it will only be possible to conclude that these children achieve satisfactory neurodevelopmental outcomes during preschool or early school age, making further studies necessary in this area [20].

### 4.4. Psychological Status of Recipients

Finally, assessing the psychological impact on these women is extremely important, as this is a procedure aimed at “improving quality of life” rather than saving lives. However, it is important to highlight the limitations of evaluating this outcome. The questionnaires used were not specifically designed for women undergoing uterine transplantation, and participants may have initially responded with a positive bias, believing it would increase their chances of inclusion in the study. Additionally, assessments conducted at 12-month intervals may overlook significant events occurring during that period. Notably, the purpose of administering the questionnaires in the initial phase was not to select participants for the study but to identify potential risks and provide support when necessary [9].

There appears to be an association between unsuccessful transplants, failed pregnancies, or the inability to achieve an LB and a decrease in the MCS score of the SF-36, as well as increased anxiety levels. Furthermore, the authors highlight that these unfavorable outcomes may cause psychological distress for couples, reinforcing the need for psychological counseling whenever necessary [9,15].

It is believed that the lower levels of anxiety and depression (HADS) and improved infertility-related QoL (FertilQol) observed in couples or women undergoing uterine transplantation, compared with couples undergoing IVF for the first time, may be related to the fact that these couples/recipients have dealt with infertility for several years, and for the first time, they may have hope of achieving a biological pregnancy [9].

Lastly, there is a curiosity regarding recent advancements in uterine transplantation: Currently, one aim is to develop bioartificial uteri derived from stem cells. This innovation would eliminate the need for uterine donation, as the organ would be genetically identical to the recipient. The same researchers have already conducted studies with partial success in animals, although they believe human application may still take two more decades [34].

Limitations: This systematic review has some limitations. First, the sample size is relatively small. This number could have been increased by including case reports. However, this would reduce the quality of the systematic review while increasing the bias of duplicate included articles. Moreover, more years and more studies conducted in this field are needed. Additionally, it is acknowledged that there is no uniformity in the criteria used across all studies (heterogeneity among studies), and there are existing significant geographical, clinical, and procedural variations among the different centers included in this review. It is important to note that this systematic review may be subject to publication bias. In other words, studies reporting positive or significant findings are more likely to be published, whereas studies with negative or inconclusive results may be underreported. Lastly, there is a notable difference in the number of LDs versus DDs, as Swedish studies, by choice, never included DDs.

## 5. Conclusions

This systematic review concludes that uterine transplantation is a highly promising technique for treating women with congenital or acquired AUFI, despite the complexity and challenges associated with the procedure. Once a functioning transplanted uterus is achieved, the results in terms of CPR and LBR per ET are very encouraging for these women. The observed obstetric risks were not higher than those reported in women who have undergone other types of solid organ transplants. No fetal malformations or neonatal complications were directly related to uterine transplantation. No significant anthropometric or neurocognitive deviations were observed in children up to three years of age. The psychological impact on recipients and couples undergoing this procedure should always be considered, with psychological support being offered whenever necessary.

## Figures and Tables

**Table 1:** Main characteristics of the included studies.

Title of the Article	Authors	Date of Publication	Platform of Publication	DOI	Country of Study	Peer-Reviewed	Type of Study	Quality of Study
Uterus Transplant in Women With Absolute Uterine-Factor Infertility. [17]	Testa G; McKenna GJ; Wall A; Bayer J; Gregg AR; Warren et al.	September/2024	PubMed	10.1001 /jama.2024. 11679	Dallas, USA	Yes	Clinical trial	Good quality (IF ≥ 5)
Uterus transplantation trial: Psychological evaluation of recipients and partners during the post-transplantation year. [9]	Järvholm S; Johannesson L; Clarke A; Brännström M.	October/2015	PubMed + Medline	10.1016/ j.fertnstert. 2015.06.038	Sweden	Yes	Observational study	Good quality (IF ≥ 5)
Three-year follow-up results of two children born from a transplanted uterus. [20]	Janota J; Orlova E; Novackova M; Chmel R; Brabec R; Pastor Z et al.	October/2023	PubMed + Medline	10.5507/ bp.2023.042	Czech Republic	Yes	Observational study	Low quality (IF < 2)
First clinical uterus transplantation trial: a six-month report. [11]	Brännström M; Johannesson L; Dahm-Kähler P; Enskog A; Mölne J; Kvarnström N et al.	May/2014	PubMed + Medline	10.1016/ j.fertnstert. 2014.02.024	Sweden	Yes	Clinical trial	Good quality (IF ≥ 5)
Psychosocial outcomes of uterine transplant recipients and partners up to 3 years after transplantation: results from the Swedish trial. [15]	Järvholm S; Dahm-Kähler P; Kvarnström N; Brännström M.	August/2020	PubMed + Medline	10.1016/ j.fertnstert. 2020.03.043	Sweden	Yes	Cohort study	Good quality (IF ≥ 5)
In vitro fertilization practice in patients with absolute uterine factor undergoing uterus transplant in the United States. [22]	Walter JR; Johannesson L; Falcone T; Putnam JM; Testa G; Richards EG et al.	September/2024	PubMed	10.1016/ j.fertnstert. 2024.04.017	USA, multicenter study (Dallas, Cleveland, and Pennsylvania)	Yes	Cohort study	Good quality (IF ≥ 5)
Reproductive, obstetric, and long-term health outcome after uterus transplantation: results of the first clinical trial. [16]	Brännström, M; Dahm-Kähler, P; Kvarnström, N; Enskog, A; Olofsson, J; Olausson, M et al.	September/2022	Scopus	10.1016/ j.fertnstert. 2022.05.017	Sweden	Yes	Clinical trial	Good quality (IF ≥ 5)
Children after uterus transplantation: 2-year outcomes from the Dallas UtErus Transplant Study (DUETS). [18]	Schulz, P; Testa, G; York, J. R; Johannesson, L.	August/2022	Scopus	10.1111/ 1471-0528. 17270	Dallas, USA (DUETS)	Yes	Cohort study	Reasonable quality (IF: 2–5)
Neonatal Outcomes after Uterus Transplantation: Dallas Uterus Transplant Study. [19]	York, J. R; Testa, G; Gunby, R. T; Putman, J. M.; McKenna, G. J; Koon, E. C et al.	April/2020	Scopus	10.1055/ s-0041- 1727212	Dallas, USA (DUETS)	Yes	Observational study	Low quality (IF < 2)
Human Uterus Transplantation from Living and Deceased Donors: The Interim Results of the First 10 Cases of the Czech Trial. [21]	Fronek, Jiri; Kristek, Jakub; Chlupac, Jaroslav; Janousek, Libor; Olausson, Michael.	February/2021	Web of Science	10.3390/ jcm10040586	Czech Republic	Yes	Clinical trial	Reasonable quality (IF: 2–5)

**Table 2:** Main outcomes of the uterine transplant technique.

Variable	Outcomes
Recipient characteristics	Mean age range: 28–31.5 years (minimum: 20 years|maximum: 38 years); MRKH syndrome: 94.0% Prior hysterectomy: 6.0%
Donor source	LD: 66.0% DD: 34.0%
Transplant success rate	Overall: 74.0% LD: 75.0% DD: 57.1%
Graft failure rate	Overall: 26.0% 72.7% due to anastomotic issues/thrombosis; others due to hemorrhagic shock, infection, chronic rejection.
Acute rejection episodes	0 episodes: 28.6% 1 episode: 35.7% ≥2 episodes: 32.1%
Reported complications	Vaginal stenosis: 71.4% Infections: 44.4% Cytopenia: 57.1% Renal toxicity: 14.3%

**Table 3:** Main pregnancy and obstetric outcomes of uterine transplantation.

Variable	Outcomes
Time from transplant to 1st ET	Mean time range: 125–390 d (minimum: 64 d|maximum: 551 d)
Pregnancy rate by viable graft	Overall: 70.3% 84.6% from LD and 15.4% from DD
LBR	Per pregnancy: 60.7–73.0% from LD and 27.0% from DD Per ET: 22.0%
CPR per ET	36.3%
LB per recipient	21.4% had 2 LB 42.9% had 1 LB 35.7% had 0 LB
Pregnancy loss rate	34.6% experience ≥ 1 pregnancy loss
Obstetric complications	Preeclampsia: 15.4% Preterm labor: 15.4% Gestational HTN: 11.5% Gestational diabetes: 11.5% Other complications: intrahepatic cholestasis, cervical insufficiency, placentation abnormalities and subchorionic hematoma (vaginal hemorrhage)

**Table 4:** Main neonatal and infant outcomes of uterine transplantation.

Variable	Outcomes
Gestational age at birth	Mean age range: 35 W and 3 d–37 W and 0 d (minimum: 30 W 6 d|maximum: 38 W 1 d) Preterm: 60.7% Full-term: 39.3%
LB per donor type	87.5% per LD 12.5% per DD
Delivery method	100% cesarean section
Apgar scores	Mean: 8 at 1st min, 9 at 5th min Range: 4–10 at 1st min, 8–10 at 5th min
Congenital anomalies	78.6% had none (*n* = 11) 21.4% had minor anomalies (*n* = 3)
Neonatal complications reported	RDS, neutrophilia, hypoglycemia, fetal lung fluid
2-month outcome	100% normal anthropometry and development parameters (*n* = 12)
6-month outcome	100% normal anthropometry parameters (*n* = 13)
18-month outcome	3 with abnormal growth 3 with mild cognitive delays
2-year outcome	4 of 5 children normal 1 with persistent communication delay
3-year outcome	100% normal growth (*n* = 2) 1 with mild expressive communication deficit.

**Table 5:** Main outcomes of physiological status in uterine transplantation recipients.

Variable	Outcomes
At the inclusion of SF-36 (PCS)	100% had a good physical quality of life
At the inclusion of SF-36 (MCS)	3 recipients below threshold for good mental QoL
At the inclusion of HADS	No significant anxiety or depression reported
At the inclusion of DAS and FertilQol	100% with good marital adjustment and infertility-related QoL
3-month outcomes	Increased stress, reduced physical function, and more pain.
1-year outcomes	Lower anxiety and depression Improved fertility QoL 100% maintained good DAS.
2-year outcomes	5 recipients with reduced PCS or MCS 2 with anxiety/depression 1 divorce
3-year outcomes	PCS stable MCS and HADS improved DAS unchanged.
4-year outcomes	100% reported good QoL and good level of marital adjustment.

## Data Availability

All data analyzed in this article are included in the manuscript and are available in a publicly accessible repository. Further inquiries can be directed to the corresponding author.

## Supplementary Materials

The following supporting information can be downloaded at: https://www.mdpi.com/article/10.3390/diseases13050152/s1, Table S1: PRISMA 2020 checklist; Figure S1: PRISMA 2020 flow diagram and PRISMA 2020 for abstracts checklist.

## Abbreviations

The following abbreviations are used in this manuscript:

AUFI	Absolute uterine factor infertility
CMV	Cytomegalovirus
CPR	Clinical pregnancy rate
d	Days
DAS	Dyadic Adjustment Scale
DOI	Digital object identifier
DD	Deceased donor
DUETS	Dallas UtErus Transplant Study
ET	Embryo transfer
FertilQoL	Fertility Quality of Life Tool
HADS	Hospital Anxiety and Depression Scale
HBP	High blood pressure
ID	Identify
IF	Impact factor
IVF	In vitro fertilization
LB	Live birth
LBR	Live birth rate
LD	Live donor
MCS	Mental component summary
MRHK	Mayer–Rokitansky–Küster–Hauser
NB	Newborn(s)
PCS	Physical component summary
PE	Preeclampsia
PRISMA	Preferred Reporting Items for Systematic Reviews and Meta-Analyses
QoL	Quality of life
RDS	Respiratory distress syndrome
SF-36	Short Form Health Survey-36
SRTR	Scientific Registry of Transplant Recipients
USA	United States of America
UTI	Urinary tract infection
W	Weeks
